# Zinc oxide nanoparticles improved chlorophyll contents, physical parameters, and wheat yield under salt stress

**DOI:** 10.3389/fpls.2022.932861

**Published:** 2022-08-03

**Authors:** Muhammad Adil, Safdar Bashir, Saqib Bashir, Zubair Aslam, Niaz Ahmad, Tasaddaq Younas, Rana Muhammad Ammar Asghar, Jawaher Alkahtani, Yheni Dwiningsih, Mohamed S. Elshikh

**Affiliations:** ^1^College of Geography and Environmental Science/Key Research Institute of Yellow River Civilization and Sustainable Development and Collaborative Innovation Center on Yellow River Civilization of Henan Province, Henan University, Kaifeng, China; ^2^Department of Agricultural Engineering, Khwaja Fareed University of Engineering and Information Technology, Rahim Yar Khan, Pakistan; ^3^Department of Soil and Environmental Sciences, Ghazi University, Dera Ghazi Khan, Pakistan; ^4^Department of Agronomy, University of Agriculture Faisalabad, Faisalabad, Pakistan; ^5^Department of Soil Science, Faculty of Agricultural Sciences and Technology, Bahauddin Zakariya University, Multan, Pakistan; ^6^Hassan Al Amir Soil Analysis, Dubai, United Arab Emirates; ^7^Department of Plant Nutrition, College of Natural Resources & Environment, Northwest Agriculture and Forestry University, Xianyang, China; ^8^Department of Botany and Microbiology, College of Science, King Saud University, Riyadh, Saudi Arabia; ^9^Department of Crop, Soil and Environmental Sciences, University of Arkansas, Fayetteville, AR, United States

**Keywords:** salinity, oxidative stress, ZnO nanoparticles, chlorophyll contents, wheat yield

## Abstract

Nanotechnology has a wide range of applications. Nanotechnology refers to the particle in nanoscale used to improve agricultural productivity and to encounter the unsolved problems conventionally. Nanostructured formulation through mechanisms, such as targeted delivery or slow/controlled release mechanisms as well as conditional release, could release their active ingredients in response to the environmental conditions and biological demands more precisely. Nanotechnology has a great potential for achieving sustainable agriculture, especially in developing countries. Salinity is among the major abiotic stresses which limits the yield and quality of global crops. Zinc (Zn) is a vital micronutrient that is mandatory for the ideal growth of plants and has proved to reduce the hazardous effects of salt stress. To counter the salinity problem, a pot experiment was conducted at wire house of the Institute of Soil and Environmental Sciences (ISES), University of Agriculture, Faisalabad, Pakistan, to observe the effects of zinc oxide (ZnO) nanoparticles (NPs) on wheat variety “Gemmieza” imported from Egypt under salt stress. Notably, 10 dS m^–1^ salinity was developed artificially, and different doses of Zn conventional fertilizer and ZnO NPs were applied to potted wheat. ZnO NPs (0.12 g pot^–1^) significantly increased the physical parameters of wheat compared to control under salt stress. Application of ZnO NPs (0.12 g pot^–1^) significantly increased chlorophyll A and B contents by 24.6 and 10%, plant height at vegetative and maturity stages by 34.6 and 37.4%, shoot and spike lengths by 30.7 and 27.6%, root fresh and dry weights by 74.5 and 63.1%, and wheat grain yield by 42.2%, respectively. ZnO NPs performed better compared to Zn conventional fertilizer under salt stress and could be used in place of Zn conventional fertilizer in salt-affected soils for attaining better crop production.

## Introduction

Wheat (*Trilicum aestivum*) is among the major cereal crop in the world (Adil et al., 2022). It fulfills the maximum food requirements by providing almost 60% of the protein and calories of a normal diet (Khalil and Jan, 2002) and is the predominant ingredient in the human diet (Arshad, 2021). In Pakistan, wheat is grown on a total area of 9.18 million hectares (Chandio et al., 2016). Demand for wheat to feed a growing population is increasing gradually, but the land under cultivation of wheat is reducing regularly due to rapid urbanization and industrialization (Salem et al., 2016). In this alarming situation, there is a dire need to improve wheat production per unit area from existing land (Kiome, 2003). In spite of the best efforts and intensive research, farmers have failed to exploit the impending yield of high-yielding wheat cultivars. One of the key reasons for low yield in Pakistan is the low use efficiency of fertilizers and salinity (Syed et al., 2021). By altering the existing fertilizers into more efficient fertilizers, this gap in the existing yield of a cultivar can be narrowed down. Millions of people and various soils around the world suffer from micronutrient deficiency affecting human health. As a result of a soil test, fertilization of soils deficient in nutrients with micronutrients separately and with nitrogen, phosphorous, and potassium (NPK) increases crop yield. As there is less synchronization between fertilizer release and crop demand during the growth period, fertilizer use efficiency by crops is nearly about 50%.

Nanotechnology has the potential to play an important role in changing agriculture and food production around the world in the coming years. The consumption of food deficient in micronutrients adversely affects human health, resulting in major health issues like reduction of growth, anemia, reproductive health, and decreased mental and physical performance (Swaminathan et al., 2013). Nanotechnology is a multidisciplinary and quickly evolving field in science and technology which involves the manufacturing, processing, and application of nano-sized associations of atoms and molecules (Tran et al., 2005). Nanoparticles (NPs) are materials with at least one dimension and a size less than 100 nm (Powers et al., 2006). Nanotechnology has a clear position in transforming agriculture and food production and the capability to modify conventional agricultural practices. Most of the agrochemicals applied to the crops do not reach the target site and are lost due to several environmental factors including leaching, drifting, hydrolysis, photolysis, and microbial degradation. The development of nanosensors can be useful to know the required amount of farm inputs such as fertilizers and pesticides. They can also detect the level of soil moisture and soil nutrients. Plants can rapidly absorb nano-fertilizers. Nano-encapsulated slow-release fertilizers can save fertilizer consumption and minimize environmental pollution. Nano-fertilizer is formulated in a controlled manner to allow the release of the nutrients according to crop needs and inhibits nutrient interaction with microorganism, water, and soil which cause immobilization of nutrients (Derosa et al., 2010). NPs can be used as fertilizers to make their release in a controlled pattern such that the nutrients are only taken up by the plant and not lost to unintentional targets like soil, water, and microorganisms. The coating of nanomaterial on chemicals, such as fertilizers and pesticides, is an important use of nanotechnology (Chen and Yada, 2011). Among different nutrients required for wheat growth, zinc (Zn) is a vital micronutrient that is mandatory for the ideal growth of wheat such as increased grain yield, and dry matter production in wheat plants by the application of Zn is observed (Modaihsh, 1997; Imtiaz et al., 2003; Ozkutlu et al., 2006). Zn is valuable to increase the yield components of wheat (Kaya et al., 2002; Singh et al., 2004) and it will be helpful in improving the water use efficiency of plants if applied in an adequate amount (Bagci et al., 2007). Genc et al. (2006) reported that Zn is useful in carrying out important functions in plant metabolism, and its deficiency causes a number of harmful effects on plant growth. Zn is an important essential element in controlling plant enzymatic systems. ZnO NPs are preferably soluble, approachable, and responsive as compared to ZnO conventional fertilizer because of its size in nanoscale and more specific surface area (Borm et al., 2006). Therefore, replacing the Zn conventional fertilizer with ZnO NPs could be helpful in increasing the availability of Zn for crop nutritional demands in Zn-deficient soils.

Zn can be directly applied to the soil in organic and inorganic forms. Due to high solubility and low cost, zinc sulfate (ZnSO_4_) is the most commonly applied inorganic source of Zn. There are numerous forms in which Zn can be applied to soils including ZnO, Zn-EDTA, and Zn-oxysulfate (Cakmak, 2008). The use of artificial ZnO NPs can be helpful in enhancing the fertilizer use efficiency and may have a positive effect on the environment, e.g., decreased use of energy and fertilizer.

Keeping in view the background considerations, an experiment was planned with the following objectives: to determine the effect of ZnO NPs on physiological parameters of wheat under saline conditions and to compare the effects of ZnO NPs and ZnSO_4_ (conventional fertilizer) under both saline and non-saline conditions on wheat.

## Materials and methods

### Collection and preparation of soil

A pot experiment was conducted at the research area of the Institute of Soil and Environmental Sciences (ISES), University of Agriculture, Faisalabad (31.4278°N, 73.0758°E), Pakistan, in 2016–2017. Soil samples were collected from the research area of ISES. The soil was air-dried and ground to pass through a 2.0-mm sieve and thoroughly mixed and homogenized. An amount of 12 kg soil was added to each pot with a height of 18 inches and 12 inches wide. Treatment calculations were made for 12 kg of soil for the pot experiment. Recommended doses of treatments were applied at the time of sowing. Irrigation was given by a hand sprayer. The plant physiological parameters were measured at vegetative and harvesting stages, while chlorophyll contents were measured at harvesting. Physical and chemical characteristics of the soil (Table 1) indicated that the soil was non-saline/sodic (ECe = 2.14 dSm^–1^) with a pH 7.35. The textural class of the soil was sandy loam and the saturation percentage of the soil was 29%.

**TABLE 1:** Physico-chemical properties of soil taken for experiment.

Texture	pH	EC (dSm^–1^)	N (%)	P (mg^–1^)	K (mg^–1^)	Zn (mg^–1^)
Sandy loam	7.2	2.14	0.052	32	160	0.51

Applied treatments were T_1_: without fertilizer application (control), T_2_: N:P:K (150:100:60 kg ha^–1^) + Salinity (10 dS m^–1^), T_3_: N:P:K (150:100:60 kg ha^–1^), T_4_: Zn Bulk (0.06 g) + N:P:K (150:100:60 kg ha^–1^) + Salinity (10 dS m^–1^), T_5_: Zn Bulk (0.12 g) + NPK (150:100:60 kg ha^–1^) + Salinity (10 dS m^–1^), T_6_: Zn Nano (0.06 g) + NPK (150:100:60 kg ha^–1^) + Salinity (10 dS m^–1^), and T_7_: Zn Nano (0.12 g) + NPK (150:100:60 kg ha^–1^) + Salinity (10 dS m^–1^).

Basal doses of NPK were applied with fully recommended doses (150:100:60 kg ha^–1^) to each pot except T_1_ (control) at the start of the experiment. The ZnO NPs were applied at the time of sowing. The sources of NPK were urea, di-ammonium phosphate, and sulfate of potash, respectively.

The experiment was planned according to a completely randomized design (CRD) under seven treatments with three replications. Eight seeds of wheat variety (Gemmieza) were sown in each pot. According to the treatment plan, Zn NPs were applied at two different levels of 0.06 g and 0.12 g on treatments (T_6_ and T_7_) and Zn fertilizer (bulk) was applied at two different levels of 0.06 g and 0.12 g (T_4_ and T_5_), whereas salinity was produced and maintained at the rate of 10 dS m^–1^ in (T_2_, T_4_, T_5_, T_6_, and T_7_). The irrigation was carried out after every 15 days and whenever required. The amount of irrigation applied was 300 ml per pot up to the vegetative stage. After the vegetative stage, the amount of irrigation was increased to 500 ml. Wheat was harvested on 15 April 2017.

### Preparation of zinc oxide nanoparticles

Zinc oxide (ZnO) NPs were prepared by the precipitation method (Hsieh, 2007) for which ZnSO_4_ (0.25 M) and NaOH (0.5 M) were used in the ratio of 1:2. ZnSO_4_ (0.25 M) was prepared by dissolving 17.97 g ZnSO_4_ in 250 ml distilled water. NaOH (0.5 M) was prepared by dissolving 5g NaOH in 250 ml distilled water. ZnSO_4_ solution was taken in a 500-ml beaker and placed on a water bath. The temperature of the water bath was maintained at 65°C. NaOH (0.5 M) solution taken in the burette was added dropwise in ZnSO_4_ solution. The pH was maintained at 5.5. The solution was stirred continuously, while the dropping speed was constant during the whole procedure. The reaction is given as follows (Sheela et al., 2012):


$${\text{2NaOH+ZnSO}}_{4}->\text{Zn}{\text{(OH)}}_{2}+{\text{Na}}_{2}{\text{SO}}_{4}$$

$$\text{Zn}{\text{(OH)}}_{2}->\text{ZnO(precipitates)}$$

The stirring process continued until fine ZnO precipitates were obtained; stirring helps in increasing the quality of precipitates. At the end of the reaction, ZnO precipitates were formed in the bottom of the flask. The effluent was discarded and precipitates were washed out with the help of ethanol or washed with distilled water two to three times and then filtered. For drying purpose, these precipitates were placed in a vacuum oven for 6 h at 60–70°C. These dried precipitates obtained in nanoscale were called “ZnO NPs.” The crystalline structure of the ZnO NPs was confirmed by the X-ray diffraction technique and the size of the nanoparticles was determined using UV-visible absorption spectroscopy by using an equation derived from the effective mass model (Talam et al., 2012). The average particle size of synthesized NPs was 53.79 nm.

Diethylenetriaminepentaacetic acid (DTPA) 1.97 g was added to 800 ml distilled water and then added 2 ml 1:1 ammonium hydroxide (NH_4_OH) to facilitate dissolution and to prevent bubbliness when bicarbonate is added to obtain 0.005 M DTPA. When most of the DTPA was dissolved, 79.06 g of ammonium bicarbonate (NH_4_HCO_3_) was added and stirred gently until dissolved. The pH was adjusted at 7.6 with 6N HCl to make 1 L volume with deionized (DI) water. An amount of 10 g air-dried soil (sieved with 2 mm mesh) was weighed into a 125-ml Erlenmeyer flask, and a 20-ml extracted solution was added. Flask was shaken for 2 h on a reciprocal shaker. The suspension was filtered out by using Whatman filter paper No. 42. According to the instructions provided for the use of the equipment, atomic absorption spectrophotometer (UNICAM 969, Unicam, Cambridge, United Kingdom) was operated. Suitable standards were run (Zn: 0, 0.2, 0.4, 0.6, 0.8, and 1 ppm) and prepared a calibration curve. Micronutrient cation was measured in the samples (soil extracts) by atomic absorption spectrophotometer using an appropriate lamp for each element. Then, micronutrient cation concentrations were recorded according to the calibration curve.

The calculation was performed according to the standard scientific procedures given by Ryan et al. (2001).

Micronutrient cation (ppm) = ppm cation from calibration curve × dilution factor

### Determination of plant parameters

Plant height was recorded at vegetative and maturity stages. The height was recorded with the help of a meter rod from the point where the stem begins to the upper tips of the shoot (vegetative stage) and spike (maturity stage) of the plant and then averaged.

Spike length was recorded at maturity of the crop. The length was recorded with the help of a meter rod from the point where the spike begins to the upper tip of the spike of the plant and then averaged.

Chlorophyll a and b were calculated according to Arnon (1949) using 500 mg of fresh leaf extracted overnight with 80% acetone and centrifuged at 10,000 rpm for 5 min. The second leaf from the apex was measured at three different positions for chlorophyll contents and then averaged.

Shoot fresh weight was measured as follows. At vegetative and maturity stages, plants per pot were harvested, and immediately their fresh weight was recorded with the help of electrical balance. Shoot dry weight was measured as follows. At vegetative and maturity stages, plants from each pot were harvested and air-dried for 2 days. Then, the samples were placed in an oven at 65°C for 1 week. After oven drying, shoot dry weight was recorded with the help of an electrical balance. Root fresh weight was measured as follows. After taking roots from pots, they were immediately weighed on an electrical balance. Root dry weight was measured as follows. Roots were air-dried for 2 days and then placed in an oven at 65°C for 1 week. After oven drying, root dry weight was recorded in grams using an electrical balance (Chyo MJ-3000) in lab. Grains and shoot samples were collected for analysis and then air-dried. After air-drying, samples were placed in an oven (EYELA, WFO-600 ND) for oven drying at 65°C. After oven drying, the samples were ground to a fine powder in a sample grinder fitted with stainless steel blades. Ground samples were stored in polythene zip bags for analysis.

Harvest index (HI) was calculated by the following formula (Sharma and Smith, 1986):

Harvest index (%) = Grain yield/Biological yield × 100.

### Statistical analysis

Data for different growth and yield attributes were collected and analyzed statistically using the software “Statistix 8.1^®^ “ version and means were compared using an least significant difference (LSD) test (Steel et al., 1997) at a 5% probability level.

## Results

### Effect of zinc oxide nanoparticle on plant height

**FIGURE 1:**
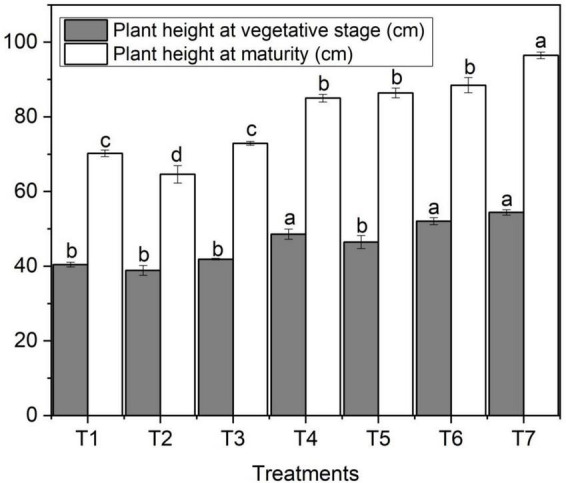
Effects of different treatments on plant heights under salinity stress. Different letters indicate a significant difference by Duncan’s multiple range tests at *p* < 0.05. Values are means ± SE (*n* = 3). T_1:_ without fertilizer application (control), T_2_: N:P:K (150:100:60 kg ha^– 1^) + Salinity (10 dS m^– 1^), T_3_: N:P:K (150:100:60 kg ha^– 1^), T_4_: Zinc Bulk (0.06 g) + N:P:K (150:100:60 kg ha^– 1^) + Salinity (10 dS m^– 1^), T_5_: Zinc Bulk (0.12 g) + NPK (150:100:60 kg ha^– 1^) + Salinity (10 dS m^– 1^), T_6_: Zinc Nano (0.06 g) + NPK (150:100:60 kg ha^– 1^) + Salinity (10 dS m^– 1^), and T_7_: Zinc Nano (0.12 g) + NPK (150:100:60 kg ha^– 1^) + Salinity (10 dS m^– 1^).

The effect of ZnO NPs on plant height varied significantly among different concentrations of applied fertilizers. Figure 1 designates the effect of foliar application of Zn NPs on wheat height. The data regarding plant height showed that ZnO NPs significantly increased plant height at vegetative and maturity stages compared to other treatments such as maximum plant height at the vegetative stage (54.4 cm) with an increase of 34.6% and at the maturity stage (96.5 cm) with an increase of 37.4% was recorded with ZnO NPs (T_7_) compared to control (T_1_) treatment (Figure 1). However, ZnO NPs (T_6_) also performed better with a plant height of 52.1 cm at the vegetative stage, followed by T_5_ (50.5 cm), and at the maturity stage (88.5 cm) and (86.5 cm); however, the control treatment showed plant heights of 40.4 cm at the vegetative stage and 70.2 cm at the maturity stage (Table 2).

**TABLE 2:** Wheat physical parameters under different doses of Zn application.

Treatment	Plant height vegetative (cm)	Plant height maturity (cm)	Shoot length (cm)	Spike length (cm)	Root dry weight (g)	Thousand grain weight (g)	Aboveground biomass yield (Mgha-1)	Grain yield (Mgha-1)	Harvest index
T_1_	40.8 c	70.2 c	62.4 d	6.5 c	1.99 c	35.3 d	12.6 b	3.51 c	0.28 b
T_2_	37.1d	65.1d	58.3d	4.5 d	1.28 d	31.2d	9.3 d	2.11d	0.22 c
T_3_	43.9 c	83.9 b	72.0 c	7.4 b	2.08 b	37.7 c	12.3 c	3.74 c	0.30 a
T_4_	50.2 b	85.0 b	74.4 c	7.4 b	2.29 b	42.3 b	12.9 b	4.91 a	0.38 a
T_5_	50.5 b	86.4 ab	73.8 c	7.0 b	2.40 ab	39.3 c	13.2 ab	3.93 c	0.29 b
T_6_	52.1 a	88.5 ab	76.1 b	8.4 a	2.97 a	43.7 b	13.5 a	4.48 b	0.33 a
T_7_	54.4 a	96.5 a	81.7 a	9.4 a	2.68 a	47.3 a	13.3 ab	4.99 a	0.35 a

T_1:_ without fertilizer application (control), T_2_: N:P:K (150: 100: 60 kg ha^–1^) + Salinity (10 dS/m), T_3_: N:P:K (150: 100: 60 kg ha^–1^), T_4_: Zinc Bulk (0.06 g) + N:P:K (150: 100: 60 kg ha^–1^) + Salinity (10 dS/m), T_5_: Zinc Bulk (0.12 g) + NPK (150: 100: 60 kg ha^–1^) + Salinity (10 dS/m), T_6_: Zinc Nano (0.06 g) + NPK (150: 100: 60 kg ha^–1^) + Salinity (10 dS/m), T_7_: Zinc Nano (0.12 g) + NPK (150: 100: 60 kg ha^–1^) + Salinity (10 dS/m). Different letters indicate the significant difference by Duncan’s multiple range tests at p < 0.05. Values are means ± SE (n = 3).

### Root fresh and dry weights

**FIGURE 2:**
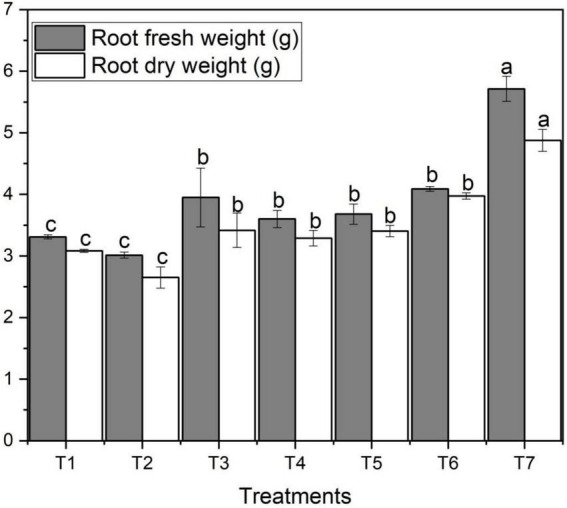
Effects of different treatments on root fresh and dry weights under salinity stress. Different letters indicate a significant difference by Duncan’s multiple range tests at *p* < 0.05. Values are means ± SE (*n* = 3). T_1:_ without fertilizer application (control), T_2_: N:P:K (150:100:60 kg ha^– 1^) + Salinity (10 dS m^– 1^), T_3_: N:P:K (150:100:60 kg ha^– 1^), T_4_: Zinc Bulk (0.06 g) + N:P:K (150:100:60 kg ha^– 1^) + Salinity (10 dS m^– 1^), T_5_: Zinc Bulk (0.12 g) + NPK (150:100:60 kg ha^– 1^) + Salinity (10 dS m^– 1^), T_6_: Zinc Nano (0.06 g) + NPK (150:100:60 kg ha^– 1^) + Salinity (10 dS m^– 1^), and T_7_: Zinc Nano (0.12 g) + NPK (150:100:60 kg ha^– 1^) + Salinity (10 dS m^– 1^).

Root fresh and dry weights varied significantly among different concentrations of ZnO NPs and Zn bulk treatments. Figure 2 designates the effect of different concentrations of foliar application of ZnO NPs on root fresh and dry weights of wheat. Figure 2 shows that salt stress decreased yield per plant and thousand-grain weight of wheat in T_2_ treatment. However, with the addition and increase in ZnO NPs concentration, root fresh and dry weights increased which ultimately increased the wheat yield. Root fresh and dry weights varied noticeably among different ZnO NPs concentrations under saline condition (Figure 2). The highest root fresh weight (5.71 g) and dry weight (4.88 g) were obtained under the highest ZnO NPs concentration (T_7_) compared to the control treatment (3.31 g) and (3.08 g), respectively; however, the lowest was observed with T_3_ (Figure 2). In both ZnO NPs treatments, with an increase in ZnO NPs from 0.06 g to 0.12 g, root fresh and dry weights also increased; in contrast, the lowest obtained under salinity with no Zn applied T_2_ treatment followed by T_4_, T_5_, T_3_, and T_6_, respectively (Table 2).

### Chlorophyll contents

**FIGURE 3:**
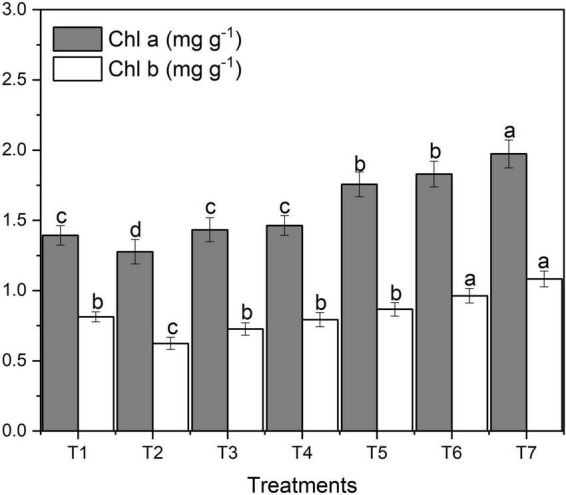
Effects of different treatments on chlorophyll contents under salinity stress. Different letters indicate a significant difference by Duncan’s multiple range tests at *p* < 0.05. Values are means ± SE (*n* = 3). T_1:_ without fertilizer application (control), T_2_: N:P:K (150:100:60 kg ha^– 1^) + Salinity (10 dS m^– 1^), T_3_: N:P:K (150:100:60 kg ha^– 1^), T_4_: Zinc Bulk (0.06 g) + N:P:K (150:100:60 kg ha^– 1^) + Salinity (10 dS m^– 1^), T_5_: Zinc Bulk (0.12 g) + NPK (150:100:60 kg ha^– 1^) + Salinity (10 dS m^– 1^), T_6_: Zinc Nano (0.06 g) + NPK (150:100:60 kg ha^– 1^) + Salinity (10 dS m^– 1^), and T_7_: Zinc Nano (0.12 g) + NPK (150:100:60 kg ha^– 1^) + Salinity (10 dS m^– 1^).

The effect of ZnO NPs on chlorophyll contents varied significantly among different concentrations of applied fertilizers. Figure 3 designates the effect of different concentrations of foliar application of ZnNPs on chlorophyll contents.

Figure 3 shows that salt stress decreased chlorophyll a, b and total content of wheat. However, with an increase in ZnO NPs concentration, chlorophyll content increased gradually. The amount of chlorophyll a and total chlorophyll varied noticeably among different ZnO NP concentrations under saline condition (Figure 3). The highest content of chlorophyll a (1.86 mg g^–1^) and b (1.03 mg g^–1^) was obtained under the highest ZnO NP concentration (T_7_) compared to control treatment (1.49 mg g^–1^) and (0.92 mg g^–1^), respectively; however, the lowest was observed with T_2_ (Figure 3). In both ZnO NP treatments, with an increase in ZnO NPs from 0.06 g to 0.12 g, the content of chlorophyll a, b and total also increased. The highest total chlorophyll content was found in ZnO NPs 0.12 g (T_7_) under salinity; in contrast, the lowest was obtained with T_2_ treatment followed by T_4_, T_1_, T_3_, and T_5_, respectively (Table 2).

### Yield per plant and 1,000 grain weight

**FIGURE 4:**
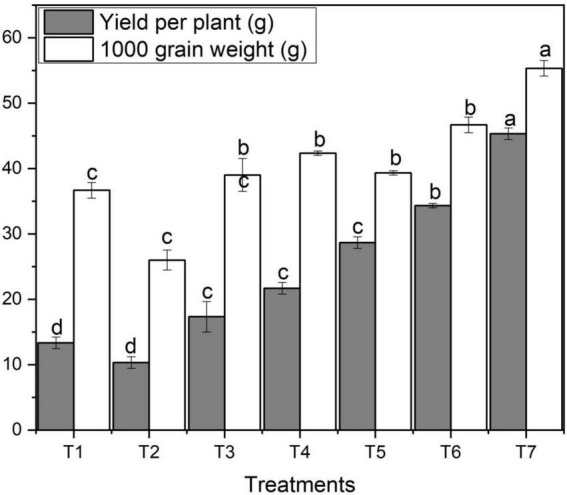
Effects of different treatments on grain yield per plant and 1,000 grain weight under salinity stress. Different letters indicate a significant difference by Duncan’s multiple range tests at *p* < 0.05. Values are means ± SE (*n* = 3). T_1_: without fertilizer application (control), T_2_: N:P:K (150:100:60 kg ha^– 1^) + Salinity (10 dS m^– 1^), T_3_: N:P:K (150:100:60 kg ha^– 1^), T_4_: Zinc Bulk (0.06 g) + N:P:K (150:100:60 kg ha^– 1^) + Salinity (10 dS m^– 1^), T_5_: Zinc Bulk (0.12 g) + NPK (150:100:60 kg ha^– 1^) + Salinity (10 dS m^– 1^), T_6_: Zinc Nano (0.06 g) + NPK (150:100:60 kg ha^– 1^) + Salinity (10 dS m^– 1^), and T_7_: Zinc Nano (0.12 g) + NPK (150:100:60 kg ha^– 1^) + Salinity (10 dS m^– 1^).

The application of ZnO NPs on grain yield per plant and thousand-grain weight varied significantly among different concentrations of ZnO NPs and Zn bulk treatments. Figure 4 designates the effect of different concentrations of foliar application of ZnO NPs on yield per plant and thousand-grain weight. Figure 4 shows that salt stress decreased grain yield per plant and thousand-grain weight of wheat. However, with the addition and increase in ZnO NPs concentration, yield per plant increased which ultimately increased thousand-grain weight. Grain yield per plant and thousand-grain weight varied noticeably among different ZnO NPs concentrations under saline condition (Figure 4). The highest yield per plant (45.33 g) was obtained under the highest ZnO NPs concentration (T_7_) compared to the control treatment (36.7g); however, the lowest was observed with T_3_ (Figure 4). In both ZnO NPs treatments, with an increase in ZnO NPs from 0.06 g to 0.12 g, yield per plant and thousand-grain weight increased (Table 2). The highest thousand-grain weight (55.3 g) was found with ZnO NPs 0.12 g (T_7_) under salinity; in contrast, the lowest was obtained with T_3_ treatment followed by T_2_, T_1_, T_5_, and T_4_, respectively.

### Grain yield and harvest index

The application of ZnO NPs on grain yield ha^–1^ and HI varied significantly among different concentrations of ZnO NPs and Zn conventional fertilizer treatments. Table 2 designates the effect of different concentrations of foliar application of ZnO NPs on grain yield ha^–1^ and HI. Table 2 shows that salt stress decreased grain yield and HI of wheat. However, with the addition and increase in ZnO NP concentration, grain yield increased which ultimately increased HI. Grain yield and HI varied noticeably among different ZnO NP concentrations under saline condition (Table 2). The grain yield (4.99 Mg ha^–1^) was obtained under the highest ZnO NP concentration (T_7_) compared to the control treatment (3.51 Mg ha^–1^); however, the lowest (2.11 Mg ha^–1^) was observed with T_2_ (Table 2). In both ZnO NP treatments, with an increase in ZnO NPs from 0.06 g to 0.12 g, grain yield and HI increased (Table 2). The highest HI (0.35) was observed in ZnO NPs 0.12 g (T_7_) under salinity; in contrast, the lowest (0.22) was obtained with T_2_ treatment.

## Discussion

Nanotechnology is a multidisciplinary and quickly evolving field in science and technology which involves manufacturing, processing, and application of nano-sized associations of atoms and molecules (Tran et al., 2005). NPs are materials with at least one dimension and a size of less than 100 nm (Powers et al., 2006). NPs can be used as fertilizers to make their release in a controlled pattern such that the nutrients are only taken up by the plant and are not lost to unintentional targets such as soil, water, and microorganisms. The coating of nanomaterial on chemicals such as fertilizers and pesticides is an important use of nanotechnology (Chen and Yada, 2011). ZnO NPs are preferably soluble, approachable, and responsive as compared to ZnO conventional fertilizer because of its size in nanoscale and more specific surface area (Borm et al., 2006). Therefore, replacing the Zn fertilizer with ZnO NPs could be helpful in increasing the availability of ZnO for crop nutritional demands in Zn-deficient soils.

The application of ZnO NPs at the time of sowing significantly increased the plant height compared to Zn bulk (Table 2) (Prasad et al., 2012).

The data regarding the effect of ZnO NPs on shoot length indicated that the application of ZnO NPs showed a better response for shoot length at the maturity stage as compared to Zn bulk (Hosseini and Maftoun, 2008).

The results for the spike length of this research were similar to the results of Rizwan et al. (2019) who reported that the spike length of wheat increased significantly by Zn NPs application. Zn bulk treatments showed less root fresh matter production than that of ZnO NPs and caused more loss of fertilizer and remained less available for plants (Ellison et al., 2013). In the case of ZnO NPs, the fertilizer was released slowly and fulfilled the crop Zn need (Trenkel, 2010), and increased the root fresh matter due to more moisture contents because both Zn and nano-sized ZnO NPs favor moisture retention in plants (Lau and Sarkar, 1984).

Zn can be directly applied to soil in both organic and inorganic forms. Due to high solubility and low cost, ZnSO_4_ is the most commonly applied inorganic source of Zn. There are numerous forms in which Zn can be applied to soils which include ZnO, Zn-EDTA, and Zn-oxysulfate (Cakmak, 2008). The use of artificial ZnO NPs can be helpful in enhancing the fertilizer use efficiency and may have a positive effect on the environment, e.g., decreased use of energy and fertilizer. Nano-fertilizer is formulated in a controlled manner to allow the release of the nutrients according to crop needs and inhibits nutrient interaction with microorganism, water, and soil which cause immobilization of nutrients (Derosa et al., 2010).

The results designated that chlorophyll contents, plant height, leaf area, shoot and root fresh and dry weights, and the concentration of Zn in soil decreased under saline conditions. The key inhibitory result of salinity on plant growth parameters and grain yield may be due to specific ion toxicity, osmotic effect, and nutritional imbalance, which results in a reduction in photosynthetic proficiency and other physiological disorders.

The critical concentration range of Zn may differ for different crop plants. Critical Zn concentration lies between 15 and 20 μg g^–1^ in young leaves of crops (Hafeez et al., 2013). However, Zn deficiency may occur at 7–30 μg g^–1^, depending upon plant species and stage of growth and development (Reuter and Robinson, 1997). Critical concentration (on a dry weight basis) in the young expended leaves for the diagnosis of Zn deficiency ranges from 11 to 14 μg g^–1^ in wheat. Zn deficiency symptoms are first appeared in the young leaves, demonstrating the low phloem movement of Zn in most of the plant species. In broad-leaf crop plants, reduced internodal length, known as resetting, is also accompanied by small leaves.

The application of Zn NPs on plant height, chlorophyll content, shoot and root dry matter, grain yield, thousand-grain weight, and Zn concentration in soil was significant under saline stress. Salt stress reduced all the above traits under salinity. Moreover, salinity also decreased the percentage of these traits. The application and increased concentration of Zn NPs alleviated the negative effects of salinity, which is revealed in increased plant height and grain yield. Less root fresh matter was produced than in treatments in which both NPK and Zn were applied in the form of ZnO NPs as Zn is an essential element for plant growth (Havlin and Benson, 2006).

Zinc oxide (ZnO) NPs made a slow release of fertilizer and enhanced the bioavailability of Zn for plants which increased plant growth, plant dry matter, and root fresh and dry matter. The treatments where Zn was not applied showed less root dry matter production than Zn applied treatment (Ellison et al., 2013). In the case of ZnO NPs, ZnO was constantly released due to coated urea and fulfilled the crop need for ZnO (Trenkel, 2010) and increased the root dry matter and grain yield due to more moisture contents because ZnO NPs favor moisture in plants (Lau and Sarkar, 1984).

Zinc (Zn) NPs showed a better response in increasing root dry matter production than control and also than other treatments in which Zn bulk treatment was applied, and this might be due to the reasons that ZnO NPs improved the fertilizer use efficiency and Zn is an essential micronutrient for plant growth (Havlin and Benson, 2006). The effect of ZnO NPs on the plant height, chlorophyll contents, shoot and root dry weights, and grain yield varied significantly between different concentrations of ZnO NPs and Zn bulk treatments. The values of all these traits for Zn NPs were greater than those for Zn bulk. In addition, there is a significant difference between the two levels of Zn NPs in terms of plant height, chlorophyll contents, and grain yield. The response of higher levels of ZN NPs was more considerable than that of low levels, which have been shown in the overall dry matter of wheat.

## Conclusion

Nanotechnology has a great potential for achieving sustainable agriculture. Zn NPs performed better compared to conventional Zn fertilizers. ZnO NPs significantly increased the physical parameters of wheat compared to control under salt stress. Application of ZnO NPs (0.12 g pot^–1^) increased chlorophyll A and B contents, plant height at vegetative and maturity stages, shoot and spike lengths, and root fresh and dry weights which ultimately increased grain yield. Although the application of conventional fertilizers enhanced wheat physical parameters under saline conditions, Zn NPs had more prominent effects on wheat physical parameters and chlorophyll contents. Therefore, in saline agricultural systems, the application of Zn NPs is a better option for increasing crop productivity.

## Data availability statement

The original contributions presented in this study are included in the article/supplementary material, further inquiries can be directed to the corresponding author.

## Author contributions

MA, ZA, and ME analyzed the data, prepared figures and/or tables, and approved the final draft. NA conceived and designed the experiments, authored or reviewed drafts of the manuscript, and approved the final draft. TY, RA, and YD performed the experiments, authored or reviewed drafts of the manuscript, and approved the final draft. JA contributed to funding acquisition. All authors contributed to the article and approved the submitted version.
